# Invasion genetics of vendace (*Coregonus albula* (L.)) in the Inari-Pasvik watercourse: revealing the origin and expansion pattern of a rapid colonization event

**DOI:** 10.1002/ece3.552

**Published:** 2013-04-12

**Authors:** Kim Præbel, Karl Øystein Gjelland, Erno Salonen, Per-Arne Amundsen

**Affiliations:** 1 Department of Arctic and Marine Biology, Faculty of Biosciences, Fisheries and Economics, University of TromsøN-9037, Tromsø, Norway; 2 Finnish Game and Fisheries Research Institute, Inari Fisheries Research and AquacultureSaarikoskentie 8, FI-99870, Inari, Finland

**Keywords:** Biological invasions, effective population size, exotic species, genetic bottleneck, rapid divergence

## Abstract

Species invasions can have wide-ranging biological and socio-economic effects and are generally unwanted by legislation. Identification of the source population as well as the ecology and genetics of both the invader population and the receiving community is of crucial importance. The rapid invasion of a small coregonid fish vendace (*Coregonus albula*) in a major northern European subarctic watercourse has resulted in a labile ecological situation in the receiving community. The ecological impact of the invasion has been thoroughly documented, but the genetics of the invasion remains to be explored. We analyzed the genetic diversity and divergence patterns among the two possible source populations from southern Finnish Lapland and three colonists populations within the Inari-Pasvik watercourse using ten microsatellite loci in order to (i) identify the most likely source of the invasion, (ii) reveal the dispersal pattern and genetic structure of the secondary expansion, and (iii) to investigate whether the initial introduction and the secondary expansion were associated with founder effects. We revealed that repeated translocation of vendace from Lake Sinettäjärvi into a tributary lake of L. Inari in 1964–1966 is the most plausible source for the invasion. Both the initial introduction and the secondary expansion were found not to be associated with significant founder effects. The secondary expansion followed a stepping stone pattern and the source and colonist populations of this expansion have undergone rapid genetic divergence within a period of 15–35 years (ca. 8–17 generations). The rapid divergence may be contributed to lack of gene flow among the source and colonist populations due to the extensive hydroelectric damming in the watercourse. Multiple introductions and substantial genetic variation in combination with the boom-and-bust population development of the species thus likely counteracted the founder effects as well as fueled the rapid establishment and expansion of this species within the Inari-Pasvik watercourse.

## Introduction

Introductions and invasions of exotic species represent a problem of global extent (Vitousek et al. 1996; Williamson 1996; Olden and Rooney 2006). Severe ecological effects may arise from invasions or possible interactions between climate change and invasions (Sandlund et al. 1999; Mooney and Hobbs 2000; Driscoll et al. 2012), and the consequences may also be associated with huge economical costs (Moyle 1986; Mack et al. 2000; Pimentel et al. 2000). During recent decades, numerous studies have addressed the ecological effects of biological invasions, particularly with respect to the consequences for the receiving communities. Negative impacts include imperilment of native species (Allan and Flecker 1993; Peterson et al. 2004; Bøhn et al. 2008), alterations of community structure and natural biodiversity (Williamson 1996; Parker et al. 1999), and a worldwide biotic homogenization (Rahel 2000, 2002; Olden and Poff 2004; Olden and Rooney 2006). However, species introductions may also be seen as large-scale ecological experiments, and provide unique insight into ecological interactions as well as population biology and genetics (Sakai et al. 2001; Shea and Chesson 2002; Sax et al. 2007; Davis 2009).

The genetic consequences and outcome of an invasion invent for the invaders and the colonist populations, that is, the correlation between propagule pressure, genetic variability of the invaders and the resulting genetic variability in the colonist populations, are not trivial. Colonist populations, that is, the populations descending from introduction events, may rapidly develop differences in morphology and phenotypes (Facon et al. 2008; Ward et al. 2012) and in ecology (Sandlund 1992; Sakai et al. 2001; Amundsen et al. 2012) as compared to the source population, but the genetic component is often unknown (e.g., Blanchet 2012). Colonist populations can give rise to secondary expansions, that may cover large geographical scales (Facon et al. 2008; Brown and Stepien 2009; Reusch et al. 2010). Such demographic expansions propel divergent adaptive pressures due to exploration of new environments (Ghalambor et al. 2007), which again may promote rapid changes in life history of the invader (Dlugosch and Parker 2008a; Amundsen et al. 2012; Gutowsky and Fox 2012). While most studies have found lower genetic variation in the colonist compared to the source populations (Henry et al. 2009; Ayres et al. 2010; but see Dlugosch and Parker 2008b for review), others show no loss of genetic variation (Novak and Mack 2005; Stepien et al. 2005; Wares et al. 2005). Some studies even find increased genetic variation in the colonist populations when they originate from multiple introductions promoting genomic admixture (Golubtsov et al. 1993; Kolbe et al. 2004; Lavergne and Molofsky 2007). The fraction of genetic variation transferred from the source populations to the invaders depends on propagule pressure (Lockwood et al. 2005; Colautti et al. 2006; Simberloff 2009), dispersal mode (Hewitt 1996; Ibrahim et al. 1996; Wilson et al. 2009), and/or route of invasion (Estoup and Guillemaud 2010). The altered selection pressure applied when individuals explore new geographical ranges also represents challenges for the invader (Lee 2002; Suarez and Tsutsui 2008). Thus, it is the combined effect of these factors that determine whether the invaders are successful in their colonization and proliferation.

**Figure 1:**
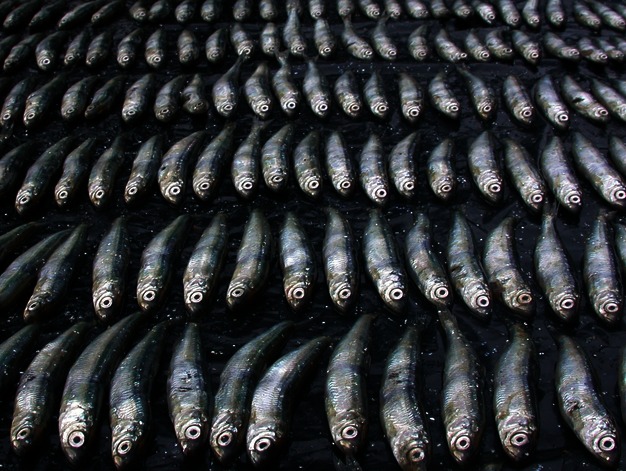
Invading vendace (*Coregonus albula*) from the Pasvik-Inari watercourse, Norway (photo: K. Ø. Gjelland).

**Figure 2:**
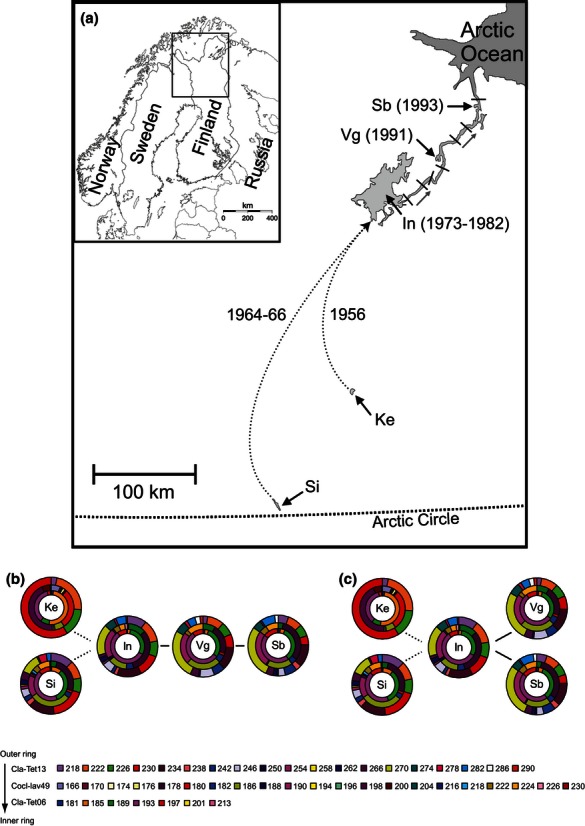
Origin and sample locations (a) and molecular genetic variation (b, c) of vendace in northern Fennoscandia. In (a) light gray arrows show the direction of drainage. Also, note that hydropower dams (indicated by black lines in the map) are located between the introduced L. Inari population (In) and the populations within the secondary expansion (Vg and Sb) limiting downstream gene flow and ruling out any upstream gene flow. The schematic illustration of the invasion process following a stepping stone (b) and normal dispersal pattern (c) are illustrated with the corresponding pie charts of the relative allelic frequencies of three representative microsatellite loci (ClaTet13, Cocl-lav49, and ClaTet06). Dashed lines indicate man-made translocations (L. Sinettäjärvi and L. Kelujärvi to L. Inari) and full black lines delineate the natural secondary expansion for the invasion. Below are the color codes for the alleles per locus. Sample locations are abbreviated according to Table 1.

The present study concerns the introduction and subsequent invasion of an exotic fish species (vendace, *Coregonus albula*, Fig. 1) into the subarctic Inari-Pasvik watershed (Norway, Finland, and Russia). The first introduction occurred in 1956, when some vendace fry were translocated for stocking purposes from Lake Kelujärvi in central Finland to the Inari Hatchery, from where they subsequently escaped into Lake Inari (Mutenia and Salonen 1992; see also Fig. 2). The second introduction occurred in 1964–1966 when vendace fry were translocated from Lake Sinettäjärvi to a small lake in the catchment area of Lake Inari. The first vendace were observed in Lake Inari in 1973. By the early 1980s, a vendace population had established throughout the lake (Mutenia and Salonen 1992), increasing to a peak abundance in 1989 (Salonen 1998, 2004). During this period a downstream invasion of vendace apparently occurred to lakes in the Pasvik watercourse, the outlet river from Lake Inari, where the species was observed for the first time in 1989 (Amundsen et al. 1999). The vendace were observed in L. Vaggatem for the first time in 1991 and in L. Skrukkebukta in 1993, and within few years the invader became an important pelagic fish species in lakes in the Pasvik watercourse (Bøhn et al. 2004, 2008). Severe ecological consequences have been documented for the receiving system, including changes in biodiversity and species composition, food web dynamics and ecosystem functioning (e.g., Bøhn and Amundsen 1998, 2001; Amundsen et al. 2003). The vendace have during the invasion entered a typical fluctuating ‘boom-and-bust’ development (Salonen et al. 2007), resulting in a destabilized aquatic ecosystem.

Although documentation of the ecological impact of the vendace invasion is accumulating, the genetics of the invasion remains to be addressed. A recent study revealed reproductive isolation between vendace in L. Inari and a downstream lake (Amundsen et al. 2012), which initiated this more detailed genetic study of the system. Because we hold detailed temporal and ecological information about the invasion process (see e.g., Mutenia and Salonen 1992; Gjelland et al. 2007; Bøhn et al. 2008) and have the special opportunity to precisely identify the source of the invasion, the system offers an excellent model system to assess ongoing genetic and evolutionary changes. We also expect the downstream invasion to be highly influenced by the seven hydropower dams that exist in the watercourse (Amundsen et al. 2012). The dams only allow unidirectional downstream gene flow that would be limited or absent during normal river flow conditions, as the fish larvae or adults would need to pass through dam turbines (see also Amundsen et al. 1999, 2012). Hence, potential factors that may limit the successful colonization in the system may relate both to founder effects and to unidirectional gene flow. Here, we used ten microsatellites to explore the genetics of the introduction and subsequent downstream migration of vendace in the watercourse by comparing the two potential source populations, the introduced population, and two downstream colonist populations originating from the secondary expansion. The main objectives of the study were to (i) identify the most likely source of the invasion, (ii) reveal the dispersal pattern and genetic structure of the secondary expansion, and (iii) to investigate whether the initial introduction and the secondary expansion were associated with founder effects.

## Materials and Methods

### Study lakes and sample collection

To identify the most likely origin of the invasion, vendace were sampled from the introduced population in L. Inari, northern Finland, and the two potential source populations, L. Sinettäjärvi and L. Kelujärvi, central Finland (Table 1; Fig. 2). Vendace were furthermore sampled from the colonist populations in L. Vaggatem and L. Skukkebukta in the Norwegian part of the Inari-Pasvik watercourse to investigate the genetics of the secondary expansion. All samples were collected in the autumn 2008 near or at the spawning grounds and consisted of >92% ripe individuals. There are seven hydropower dam constructions located below L. Inari (Fig. 2). More detailed descriptions of L. Inari and the Pasvik watercourse are given by Mutenia and Salonen (1992) and Amundsen et al. (1999), respectively.

**Table 1:** Locations and codes samples included in the genetic analysis of the northern European vendace invasion (Fig. 2), with latitude and longitude (position), sample date, water drainage, lake size and height of location in water course, date of translocation (for L. Kelujärvi and L. Sinettäjärvi to L. Inari) or first observation on locality (for L. Inari, L. Vaggatem and L. Skrukkebukta), secchi depth, and sample size (*N*)

Lake	Code	Position	Sample date	Water course (Ocean)*	Area (km^2^)	Height (m)	Year (Observed)	Secchi depth (Max depth, m)	*N*
L. Kelujärvi	Ke	67°28′N, 27°4′E	14.11.2008	Kemijoki (BS)	9	187	1956	2 (10)	52
L. Sinettäjärvi	Si	66°35′N, 25°25′E	25.10.2008	Kemijoki (BS)	9	97	1964–1966	3 (40)	58
L. Inari	In	69°10′N, 27°55′E	30.10.2008	Inari-Pasvik (AO)	1102	118	1973	5 (95)	55
L. Vaggatem	Vg	69°13′N, 29°14′E	09.09.2008	Inari-Pasvik (AO)	34	52	1991	3 (30)	57
L. Skukkebukta	Sb	69°33′N, 30°7′E	16.09.2008	Inari-Pasvik (AO)	7	21	1993	6 (38)	54

* BS, Baltic Sea; AO, Arctic Ocean.

### Microsatellite DNA amplification and genotyping

Genomic DNA was extracted from gill filaments by E-Z96 Tissue DNA Kit (OMEGA Bio-tek, Norcross, GA) following the manufacturer instructions. The individuals were genotyped at 10 microsatellite loci (Table S1) arranged in three multiplex polymerase chain reactions (PCR) following a previously described protocol (Præbel et al. in press). The PCR products were separated on an ABI 3130 XL Automated Genetic Analyzer (Applied Biosystems, Foster City, CA) and alleles scored in the GeneMapper 3.7 software (Applied Biosystems). After the first validation of the genotypes, 3–4% of the individuals within each population were re-extracted and rerun at all ten loci. The genotypes resulting from the initial run and the rerun were manually compared for all individuals to rule out miss-scoring of alleles. If any doubt occurred in this comparison the samples were re-extracted and rerun at all loci to obtain a consensus genotype. The samples were finally screened for abnormalities (null alleles, scoring errors, etc.) in the software MICRO-CHECKER 2.2.3 (Van Oosterhout et al. 2004), using 1000 bootstrap replications to generate the expected homozygote and heterozygote allele size difference frequencies.

### Statistics

The within population genetic variation indices; number of alleles (*N*_A_), expected (*H*_e_) and observed (*H*_o_) heterozygosity, and the fixation index (*F*_IS_) were estimated in GenAlEx 6.41 (Peakall and Smouse 2006; Table S1). Deviations from Hardy–Weinberg equilibrium (HWE) for each population and locus and linkage disequilibrium (LD) among loci and among loci over all populations were tested by exact tests (Guo and Thompson 1992) using GENEPOP 4.0 (Rousset 2007). The tables of pair-wise *P-*values from the LD and HWE tests were corrected for multiple comparisons by sequential Bonferroni corrections (BFCs) following Rice (1989). Allelic (*N*_RA_) and private allelic richness (*N*_RPA_) per population were determined, accounting for differences in sample-sizes, using the rarefaction procedure for the smallest sample size (100 genes) as implemented in the software HP-RARE 1.0 (Kalinowski 2005).

To infer which of the two initial vendace stockings (L. Sinettäjärvi or L. Kelujärvi) in L. Inari that led to successful colonization of the Inari-Pasvik watercourse, a neighbor-joining tree was build using Nei et al. (1983) genetic distance (*D*_a_) and nodes were tested for robustness by 1000 bootstraps using Populations 1.2.32 (Langella 2005) and viewed in TREEVIEW (Page 1996). A sample of native vendace (*N* = 20) obtained from northern Germany was used to assist the clustering of the neighbor-joining tree. Genetic divergence between the possible source populations and the introduced L. Inari population as well as among the colonist populations were estimated by pair-wise *F*_ST_ (Weir and Cockerham 1984) values and tested for statistical significance (10,000 permutations) using ARLEQUIN 3.5.1.2 (Excoffier and Lischer 2010). The table of *P-*values for the pair-wise *F*_ST_ values was corrected for multiple comparisons by BFCs following Rice (1989). Bayesian clustering as implemented in STRUCTURE 2.3.2 (Pritchard et al. 2000; Hubisz et al. 2009), was used to provide another estimate of origin and population structure of the data. We used a model assuming admixture and correlated allele frequencies between K populations (Burn-ins of 100,000 replications and 300,000 Markov chain Monte Carlo (MCMC) replicates). Sampling locations were used as a priori information to assist the structuring (the LOCPRIOR model) as recommended for weak signals of structuring (Hubisz et al. 2009). All runs were replicated 10 times at each *K* = 1–5 to confirm consistency of log-likelihood probabilities. The most likely (highest ln Pr(*X*|*K*)) grouping was visualized using STRUCTURE HARVESTER (Earl and vonHoldt 2012). Structuring of the vendace populations were also estimated by principal component analysis (PCA). The ordination of the individuals was performed in the program GenAlEx 6.41 (Peakall and Smouse 2006). Finally, we investigated whether shared private alleles could be identified in pair-wise comparisons of the source L. Kelujärvi and/or L. Sinettäjärvi and the introduced population (L. Inari), as well as among populations within the watercourse which would support any link found in the population structuring approach. The rarefaction procedure implemented in ADZE 1.0 (Szpiech et al. 2008) were used to estimate the pair-wise shared private allelic richness across loci for the possible source populations and the introduced population (L. Inari) and for the secondary expansion within the water course (i.e., between L. Inari-L. Vaggatem, L. Inari-L. Skrukkebukta, and L. Vaggatem-L. Skrukkebukta). All estimates were performed using a standardized sample size corresponding to the smallest sample (100 genes).

We tested whether the initial stocking and the subsequent invasion was associated with founder effects by testing for genetic bottlenecks among the populations using the software BOTTLENECK 1.2.02 (Cornuet and Luikart 1996). Population bottlenecks will cause a temporarily imbalance in the mutation-drift equilibrium, where additions of new alleles via mutation are balanced by the loss of alleles via drift (Luikart and Cornuet 1998). We utilized this assumption to identify situations where the actual sample heterozygosity exceeds a permuted equilibrium heterozygosity as expected under a mutation-drift equilibrium. If the actual heterozygosity exceeds the equilibrium heterozygosity it is indicative of a recent population bottleneck (Cornuet and Luikart 1996). We used 1000 coalescent simulations and assumed a two-phased model of mutation (TPM) and the more conservative step-wise mutation model (SMM). The statistical significance of the deviations at equilibrium and observed heterozygosities were tested with Wilcoxon signed-rank tests. We also tested for genetic signatures of founder effects following the invasion and the secondary expansion, by estimates of changes in the effective population size (*N*_e_). Changes in the effective population size will indicate the relative contribution of genetic drift (Hedrick 2000). *N*_e_ was estimated for all populations using OneSamp 1.1 (Tallmon et al. 2008). This software uses approximate Bayesian computation to estimate variance *N*_e_ from summary statistics that are related to *N*_e_. We used prior upper and lower bounds for *N*_e_ of 2–1000 and 10,000 replications to generate the 95% credible intervals. Finally, we correlated the pair-wise *F*_ST_'s and the difference in expected heterozygosity between the source population and Inari-Pasvik populations (Δ*H*_e_) with geographic distance and years from translocation to occurrence. This was performed to reveal spatial and temporal patterns of the mechanism underlying the founder event and to estimate the time of the possible expansion from L. Inari downstream the Pasvik watercourse. We expected increased differentiation (pair-wise *F*_ST_'s) and difference in genetic diversity (Δ*H*_e_) the longer the distance and time since divergence. The correlation of genotype data and geographical distance (physical divergence) from the source population to each of the populations in the Inari-Pasvik watercourse were compared with the corresponding correlations for the three possible expansion times. The time from introduction to the founding of the L. Inari population was in all estimates set to 8 years, whereas the expansion time from L. Inari downstream the watercourse was tested for initial sighting of vendace larvae (1973), total cover of lake (1980), and peak (1989) (Table S2). We used partial Mantel tests as implemented in GenAlEx 6.41 (Peakall and Smouse 2006), to test these scenarios, using 9999 permutations to obtain significance.

## Results

### Genotyping, validation, and quality control of genotypic data

We did not identify any mismatch between the original individual multilocus genotypes and the re-extracted 3–4% replicates within the present dataset. Heterozygote deficits were indicated by MICRO-CHECKER at BWF1 (L. Vaggatem, L. Skrukkebukta), C2-157 (L. Skrukkebukta), Cocl-lav06 (L. Sinettäjärvi, L. Inari, L. Vaggatem, and L. Skrukkebukta) Cocl-lav10 (L. Sinettäjärvi, L. Inari, L. Vaggatem, and L. Skrukkebukta), Cocl-lav49 (L. Skrukkebukta), ClaTet06 (L. Kelujärvi), ClaTet13 (L. Sinettäjärvi and L. Skrukkebukta) all indicated as caused by the presence of null alleles. To test whether the loci with potential null alleles may affect the results the STRUCTURE analyses were performed as described in the Material and Methods but without the loci showing the most heterozygote deficits across populations (BWF1, Cocl-lav06, and Cocl-lav10). Neither the number of inferred clusters nor the population structure was found to differ when comparing the results with and without these loci (Figs. S1 vs. 4). Given the relatively few loci used in the study we therefore maintained all loci in the full analysis to ensure statistical power.

### Genetic variation

The standard indices of within population genetic variation are given in Tables 2 and S1. We discerned 124 alleles among the ten microsatellite loci assayed in the five studied vendace populations, with a within population variation of 47 to 89 alleles. Mean number of alleles (*N*_A_) per locus per population varied from 2 to 25 and expected (*H*_e_) and observed heterozygosity (*H*_o_) per locus per population varied from 0.019 to 0.916 and 0.019 to 0.895, respectively. All populations, except L. Kelujärvi, showed significant departures from HWE associated with heterozygote deficits. Linkage disequilibrium was identified for 4 out of 45 pair-wise locus tests across populations but all returned non-significant after sequential BFC. The individual locus tests displayed 18 out of 50 and 7 out of 50 significant deviations from HWE before and after BFCs, respectively (Table S1), and 2 to 9 significant LD's within each population (only three significant after BFC, all in L. Sinettäjärvi).

**Table 2:** Summary genetic statistics of the vendace populations included in the study

Sample	*F*_IS_	*H*_e_	*H*_o_	*N*_A_	*N*_RA_	*N*_RPA_	TPM	SMM	*N*e
Ke	0.0772	0.400	0.369	47	4.6	0.79	0.084	0.003 (d, 0.002)	55 (38–153)
Si	0.1133*	0.614	0.544	89	8.6	0.97	0.322	0.019 (d, 0.009)	234 (130–988)
In	0.0870*	0.597	0.546	89	8.8	0.73	0.193	0.007 (d, 0.003)	250 (150–746)
Vg	0.0849*	0.596	0.546	81	7.9	0.11	0.625	0.019 (d, 0.009)	150 (93–422)
Sb	0.1664*	0.607	0.506	79	8.4	0.58	0.275	0.003 (d, 0.002)	200 (112–703)

The coefficient of inbreeding (*F*_IS_), genetic diversity estimates (*H*_e_/*H*_o_), total number of alleles (*N*_A_), mean allelic (*N*_RA_), and private allelic richness (*N*_RPA_). The *P*-values for the Bottleneck test are given (TPM and SMM) and significant values (SMM) were only found significant for heterozygote deficit (denoted with d and the corresponding *P*-value for the exact test). The effective population sizes (*N*e) with credible intervals are given. Sample codes as in Table 1.

* Significant heterozygote deficit (positive *F*_IS_) estimated by Hardy–Weinberg exact tests.

The allelic richness were almost double in L. Sinet-täjärvi, L. Inari, L. Vaggatem, and L. Skrukkebukta (*N*_RA_ = 7.9–8.8) compared to L. Kelujärvi (*N*_RA_ = 4.6) (Table 2). Private allelic richness varied between *N*_RPA_ = 0.11–0.97 with the lowest *N*_RPA_ found in the lower Pasvik river lakes. *H*_e_ and *H*_o_ were also higher in L. Sinettäjärvi, L. Inari, L. Vaggatem, and L. Skrukkebukta (*H*_e_ = 0.596–0.614; *H*_o_ = 0.506–0.546) compared to L. Kelujärvi (*H*_e_ = 0.400; *H*_o_ = 0.369) (Table 2).

### Identifying the source for the vendace invasion and population structure within the secondary expansion

**Figure 3:**
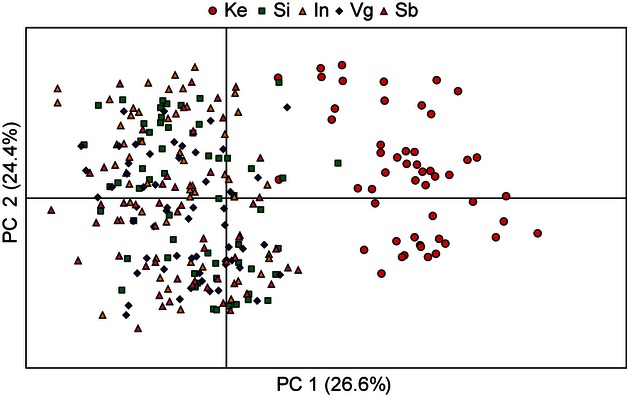
Principal component analysis (PCA) plot of the genetic structuring among the five vendace populations. PC1 and PC2 explain 26.6% and 24.4% of the total variation, respectively. Sample abbreviations as in Table 1.

The pair-wise *F*_ST_ estimates of genetic differentiation between the two possible source populations (L. Kelujärvi/L. Sinettäjärvi) and L. Inari suggest that L. Sinettäjärvi was the source population as this population pair display the lowest, although significant, differentiation (L. Kelujärvi vs. L. Inari, *F*_ST_ = 0.158, *P* < 0.0001; L. Sinettäjärvi vs. L. Inari, *F*_ST_ = 0.011, *P* = 0.0027; Table S3). L. Kelujärvi moreover appeared to be the most isolated population of the five populations studied (*F*_ST_ = 0.141–0.158; *P* < 0.0001). Within the Inari-Pasvik watercourse, the introduced population (L. Inari) was significantly different from the populations from the secondary expansion (L. Vaggatem and L. Skrukkebukta), whereas the populations from L. Vaggatem and L. Skrukkebukta could not be significantly discriminated (Table S3). The sequential BFCs did not change the significance level for any of *P*-values from the pair-wise *F*_ST_ estimates. The PCA plot revealed groupings of (i) L. Kelujärvi and (ii) L. Sinettäjärvi, L. Inari, L. Vaggatem, and L. Skrukkebukta (Fig. 3). Each axis, PC1 and PC2, explained 26.6% and 24.4% of the total variation, respectively.

**Figure 4:**
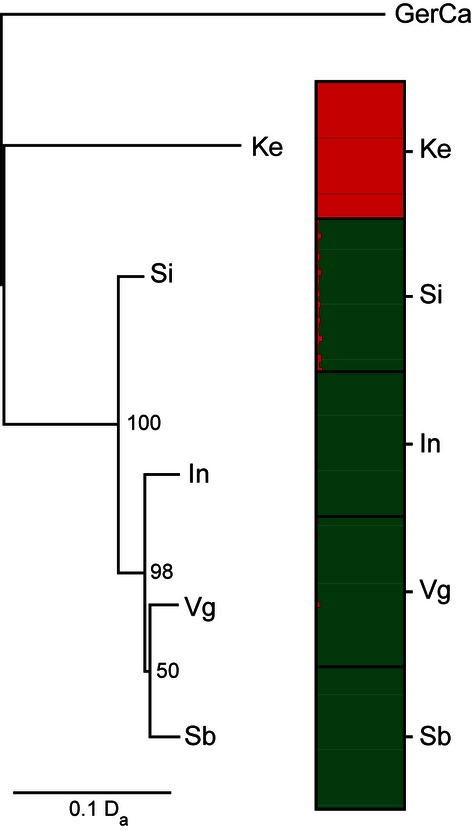
Genetic structure among L. Kelujärvi (Ke), L. Sinettäjärvi (Si), L. Inari (In), L. Vaggatem (Vg), and L. Skrukkebukta (Sb) as revealed by a neighbor-joining trees using Nei et al. (1983) genetic distance (*D*_a_) and Bayesian clustering of all individuals using STRUCTURE (Hubisz et al. 2009) assuming two genetic clusters of individuals (*K* = 2). Only bootstrap resampling percentages above 50 are shown for the neighbor-joining tree. In the STRUCTURE analysis black lines separate individuals from different sampling sites (labeled right) and each individual is represented by a thin horizontal line, which is partitioned into *K-*colored segments representing individual's estimated membership fractions in *K* clusters. The absolute mean values of ln Pr(*X*|*K*) are plotted for *K* = 1–5 in supplementary Fig. S2.

The Bayesian clustering revealed that the individuals could be partitioned into two genetic clusters (*K* = 2; ln Pr(*X*|*K*) ± SD = −7209 ± 1; Fig. S2), separating L. Kelujärvi into one and L. Sinettäjärvi, L. Inari, L. Vaggatem, and L. Skrukkebukta into the other cluster (Fig. 4). The combined approach of a neighbor-joining tree with Nei et al. (1983) genetic distance (*D*_a_) provided support for L. Sinettäjärvi being the most likely source for the vendace in the Inari-Pasvik watercourse. Moreover, the neighbor-joining tree also provided support for divergence between the founder population of L. Sinet-täjärvi and the introduced population in L. Inari (bootstrap support of 100%) as well as between L. Inari and the populations from the secondary expansion (L. Vaggatem and L. Skrukkebukta, 98%), which is also supported by the low, but significant *F*_ST_ values. Finally, the Bayesian clustering and the neighbor-joining tree grouped L. Vaggatem and L. Skrukkebukta together supporting the non-significant *F*_ST_ value estimated between these populations.

**Figure 5:**
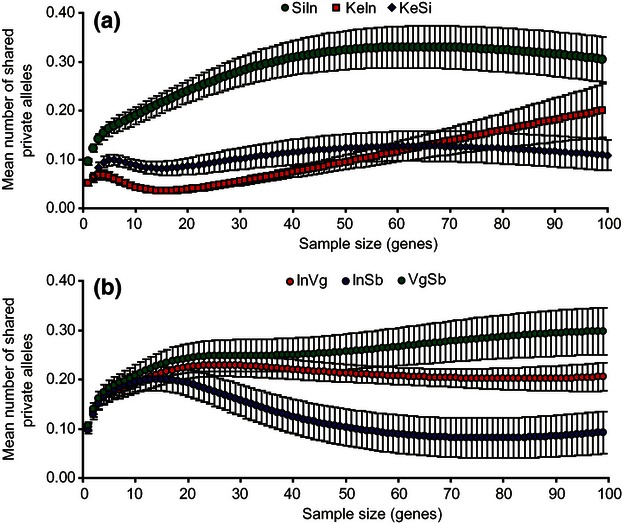
The mean number of shared private alleles private to location pairs of L. Kelujärvi-L. Inari (KeIn), L. Sinettäjärvi-L. Inari (SiIn), and L. Kelujärvi-L. Sinettäjärvi (KeSi) (a) and within the Inari-Pasvik watercourse (L. Inari-L. Vaggatem, InVg; L. Inari-L. Skrukkebukta, InSb; and L. Vaggatem-L. Skrukkebukta, VgSb) (b) as a function of standardized sample sizes (in genes). Error bars represent ±SEM across the 10 microsatellite loci.

A higher mean number of shared private alleles were found within the L. Sinettäjärvi-L. Inari pair compared to the L. Kelujärvi-L. Inari pair (Fig. 5a). The mean number of private alleles shared by the L. Kelujärvi-L. Sinettäjärvi pair resembled that of the L. Kelujärvi-L. Inari pair, supporting the conclusion that L. Sinettäjärvi is the source population for the invasion. For the secondary expansion the pair-wise comparison of mean number of shared private alleles show that the L. Inari-L. Vaggatem pair share fewer private alleles than the L. Vaggatem-L. Skrukkebukta pair, with the L. Inari-L. Skrukkebukta pair sharing the lowest number of private alleles (Fig. 5b). This suggests a stepping-stone dispersal where L. Inari vendace colonized L. Vaggatem and the population in L. Vaggatem subsequently founded the population in L. Skrukkebukta (Fig. 2b). Some specific alleles (e.g., allele 270 at ClaTet13, allele 190 at Cocl-lav49, but see Fig. 2b) were also observed only in the L. Sinettäjärvi, L. Inari, L. Vaggatem, and L. Skrukkebukta populations and display an expanding pattern, while others (e.g., allele 189 at ClaTet06) were present in all populations but in much higher/lower frequencies in L. Kelujärvi compared to the other four lakes (Fig. 2b).

### Founder effects and patterns of spatial and temporal genetic variation

None of the populations showed significantly higher expected heterozygosity (*H*_e_) than equilibrium heterozygosity (*H*_eq_) in the analysis of bottlenecks assuming a TPM. Similar results were revealed when assuming a SMM. All estimates of effective population sizes were associated with overlapping CIs (Table 2). However, the estimate of L. Sinettäjärvi and L. Inari did not differ in magnitude, supporting that the introduction of vendace into the system was associated with limited founder effects (drift). A decreasing trend in effective population size (although all CIs overlapped) was revealed from L. Inari to L. Skrukkebukta suggesting an increased influence of drift and restricted gene flow during the expansion.

All spatial and temporal correlations of genotypes were found to be significant (Table 3). The period of peak abundance of vendace (1989) in L. Inari also correlate the best with patterns of genetic expansion (Δ*H*_e_), suggesting that the secondary expansion downstream the watercourse occurred late in the invasion process and that it was associated with genetic constrains. This is also in accordance with the observation of vendace in catches in Pasvik; vendace were first observed in the upper Pasvik in 1989, and then successively downstream the watercourse (Vaggatem in 1991 and Skrukkebukta in 1993; Amundsen et al. 1999).

**Table 3:** Spatial and temporal correlations of pair-wise differences (*F*_ST_) and differences in expected heterozygosity (Δ*H*_e_) using partial Mantel tests

	*F*_ST_		Δ*H*_e_	
		
	*R*_xy_	*P*	*R*_xy_	*P*
Geographical distance	0.548	0.041	−0.987	0.040
Time_Initial_observation_	0.778	0.045	−0.623	0.040
Time_Total_coverage_	0.682	0.045	−0.817	0.042
Time_Peak_abundance_	0.483	0.042	−0.912	0.041

The coefficient of regression (*R*_xy_) and the corresponding *P*-value (9999 permutations) are given. Geographical distances and expansion times used for correlations are given in Table S2. Δ*H*_e_, Pair-wise difference in heterozygosity between L. Sinettäjärvi and the populations within the Inari-Pasvik watercourse.

## Discussion

### Primary source of the Inari-Pasvik vendace invasion

There were two possible sources for the introduction and subsequent downstream invasion of vendace in the Inari-Pasvik watercourse (Mutenia and Salonen 1992; Salonen 1998). Multilocus microsatellite data and several statistical approaches clearly identified L. Sinettäjärvi vendace as the most likely founder population of Inari-Pasvik vendace. The low, but significant, pair-wise *F*_ST_ value found between the vendace populations of L. Sinettäjärvi and L. Inari indicates that these populations have diverged recently. In comparison, the *F*_ST_ value between L. Kelujärvi and L. Inari was high and represents the typical level of genetic divergence reported between other native postglacial coregonid populations in northern Fennoscandia (Østbye et al. 2006; Saisa et al. 2008; Præbel et al. in press). Moreover, support for L. Sinettäjärvi being the source population for the invasion was found in the combined approach of a neighbor-joining tree and Bayesian clustering, where genetic homogeneity was shown for L. Sinettäjärvi and the Inari-Pasvik watercourse populations whereas L. Kelujärvi formed its own cluster. Bayesian clustering and neighbor-joining trees have successfully been used in other studies for inference of routes and sources of biological invasions (see review by Estoup and Guillemaud 2010), and supportive results, as seen herein, provide strong evidence for the revealed pattern. L. Sinettäjärvi and L. Inari also shared a higher number of private alleles than L. Kelujärvi and L. Inari, which provide additional support for L. Sinettäjärvi being the founders, as time since introduction is too short for many new private alleles to accumulate. Addressing the number of shared private alleles between pairs of populations has been used in several other studies to identify the origin of invasions and migrations (e.g., Szpiech et al. 2008; Bell and Matocq 2011). It is also worth considering that the similarity in genetic variation of L. Sinettäjärvi and L. Inari provide additional support for this origin, as biological invasions very seldom are associated with an increase in genetic variation in the introduced population compared to the source population (Nei et al. 1975; Dlugosch and Parker 2008b). Thus, taken together, there is compelling evidence that the second introduction of vendace in 1964–1966 from L. Sinettäjärvi represents the source of the vendace invasion and establishment in the Inari-Pasvik watercourse.

### Founder effects in the initial Inari-Pasvik vendace invasion

Multiple introductions have been shown to retain genetic diversity (Kolbe et al. 2004; Facon et al. 2008; Geller et al. 2010) and thus increase the likelihood of successful colonization and demographic expansion of the invader (e.g., Dlugosch and Parker 2008b; Shine 2012). In the present study the successful source population for the invasion was identified as the one that was introduced several times in 1964–1966 to a small lake upstream L. Inari. The population that apparently did not succeed in establishing was introduced to L. Inari by escapees from a local hatchery (Mutenia and Salonen 1992). This implies that the propagule pressure for this source likely has been smaller and/or that the population was presumably less adapted for the watercourse compared to the repeated introductions from L. Sinettäjärvi. In fact, the results show that the L. Inari population has similar allelic richness and heterozygosity to the source population. We did not identify loss of genetic variation via genetic bottlenecks for the introduced L. Inari population, which have been shown to be the case for other biological invasions (e.g., Tsutsui et al. 2000; Henry et al. 2009; Ayres et al. 2010). Similar effective population size estimates in the L. Sinettäjärvi and Inari-Pasvik populations also suggested little genetic drift. Thus, the initial founding event of vendace in the Inari-Pasvik watercourse may be associated with no or limited founder effects. However, we show that the source and the invasive populations are genetically different, which suggest that genetic drift actually has played a role in the establishment of the colonists, since time from introduction is too short for mutations to have accumulated across the microsatellite loci in high enough frequencies to have effect on the result.

### Dispersal pattern and genetic structure of the secondary expansion

The results revealed that the populations resulting from the secondary expansion downstream from L. Inari have diverged genetically from the introduced population in a stepping stone pattern. Such a dispersal pattern maintains genetic variation and limits divergence compared to other expansion patterns (e.g., Ibrahim et al. 1996; Reusch et al. 2010; Tonione et al. 2011), due to the allelic patchiness and likely subsequent gene flow among demes. The populations within the secondary expansion have become genetically distinguishable from the L. Inari population in about 18 years/9 generations (maximum 35 years/17 generations if the Pasvik populations were founded already in 1973 at the time of first observation in L. Inari, but this scenario appears highly unlikely from the observed occurrences of vendace in the watercourse; see Amundsen et al. 1999). This is remarkable, especially because the high genetic variation and large consensus and effective population size of the colonist population effectively should counteract genetic divergence. In marine fish species, such as herring (*Clupea harengus*), capelin (*Mallotus villosus*), and Atlantic cod (*Gadus morhua*), it is well known that large population sizes with high genetic variation and no obvious barriers to gene flow effectively hamper the build up of reproductive isolation (Knutsen et al. 2003; Mariani et al. 2005; Præbel et al. 2008; but see review by DeWoody and Avise 2000). The major difference and the most likely explanation for the rapid divergence observed among the vendace populations within the Inari-Pasvik watercourse therefore appears to be the presence of several hydroelectric dam constructions between the L. Inari population and the two downstream populations from the secondary expansion (i.e., L. Skrukkebukta). These dams may effectively hamper downstream gene flow and promote the build up of reproductive isolation during the expansion.

### Adaptive and evolutionary changes in the invader – perspectives

Identifying the source population and reconstructing the routes of biological invasions are crucial for handling and managing invasive species, as well as for gaining knowledge of the ecology behind successful colonization events. However, invasions also offer the possibility to study evolutionary processes such as natural selection and life history changes as they unfold. For example, invaders may evolve in response to an altered selection regime compared to their native range (Facon et al. 2006; Sax et al. 2007; Bacigalupe 2009; Shine 2012). The Inari-Pasvik watercourse is 300–380 km north of L. Sinettäjärvi, but at similar altitude. Thus, the summer is shorter and colder, and the ice-covered period longer. Especially, temperature is an important driver for adaptive changes as it will affect, for example, egg incubation time, growth, physiological processes (Q10), and general individual fitness (e.g., Mooney and Hobbs 2000). In addition, r-strategists are often favored during biological invasions (Lodge 1993; Facon et al. 2006), and our earlier study suggests that these invasive populations in the secondary expansion areas have speeded up their life history via growth and age at sexual maturity (Amundsen et al. 2012). These changes happened within a decade and the system therefore apparently represents an excellent example of rapid adaptive evolution. Future studies of this system may therefore gain valuable insights by identifying adaptive phenotypic traits (transcriptome) and the genetic basis for local adaptation (e.g., using phenotypic QTLs) (see e.g., Bernatchez et al. 2010). Moreover, insights would be gained from investigating variation at genes and genomic blocks related to important life history parameters such as growth, metabolism, and disease resistance, to infer adaptive changes or patterns of plasticity among the Sinettäjärvi-Inari-Pasvik populations.

## Supporting Information

**Table S1.** Summary statistics for each locus and population in the source and invader vendace (*Coregonus albula*) populations from northern Europe.

**Table S2.** Geographical distances and expansion times of vendace populations used in the partial Mantel test.

**Table S3.** Estimates of genetic differentiation (*F*_ST_) between the five populations of vendace in northern Europe.

**Figure S1.** Summary of results from STRUCTURE for inferring K populations among the tested vendace populations when omitting three loci that may be associated with null alleles.

**Figure S2.** Summary of results from STRUCTURE for inferring K populations among the tested vendace populations.

## References

[b1] Allan JD, Flecker AS (1993). Biodiversity conservation in running waters. Bioscience.

[b2] Amundsen PA, Staldvik FJ, Reshetnikov YS, Kashulin N, Lukin A, Bøhn T (1999). Invasion of vendace *Coregonus albula* in a subarctic watercourse. Biol. Conserv.

[b3] Amundsen PA, Bøhn T, Popova OA, Staldvik FJ, Reshetnikov YS, Kashulin NA (2003). Ontogenetic niche shifts and resource partitioning in a subarctic piscivore fish guild. Hydrobiologia.

[b4] Amundsen PA, Salonen E, Niva T, Gjelland KO, Præbel K, Sandlund OT (2012). Invader population speeds up life history during colonization. Biol. Invasions.

[b5] Ayres RM, Pettigrove VJ, Hoffmann AA (2010). Low diversity and high levels of population genetic structuring in introduced eastern mosquitofish (*Gambusia holbrooki*) in the greater Melbourne area, Australia. Biol. Invasions.

[b6] Bacigalupe LD (2009). Biological invasions and phenotypic evolution: a quantitative genetic perspective. Biol. Invasions.

[b7] Bell KC, Matocq MD (2011). Regional genetic subdivision in the Mohave ground squirrel: evidence of historic isolation and ongoing connectivity in a Mojave desert endemic. Anim. Conserv.

[b8] Bernatchez L, Renaut S, Whiteley AR, Derome N, Jeukens J, Landry L (2010). On the origin of species: insights from the ecological genomics of lake whitefish. Philos. Trans. R. Soc. B Biol. Sci.

[b9] Blanchet S (2012). The use of molecular tools in invasion biology: an emphasis on freshwater ecosystems. Fish. Manage. Ecol.

[b10] Bøhn T, Amundsen PA (1998). Effects of invading vendace (*Coregonus albula* L.) on species composition and body size in two zooplankton communities of the Pasvik river system, northern Norway. J. Plankton Res.

[b11] Bøhn T, Amundsen PA (2001). The competitive edge of an invading specialist. Ecology.

[b12] Bøhn T, Sandlund OT, Amundsen PA, Primicerio R (2004). Rapidly changing life history during invasion. Oikos.

[b13] Bøhn T, Amundsen P-A, Sparrow A (2008). Competitive exclusion after invasion?. Biol. Invasions.

[b14] Brown JE, Stepien CA (2009). Invasion genetics of the Eurasian round goby in North America: tracing sources and spread patterns. Mol. Ecol.

[b15] Colautti RI, Grigorovich IA, MacIsaac HJ (2006). Propagule pressure: a null model for biological invasions. Biol. Invasions.

[b16] Cornuet JM, Luikart G (1996). Description and power analysis of two tests for detecting recent population bottlenecks from allele frequency data. Genetics.

[b17] Davis MA (2009). Invasion biology.

[b18] DeWoody JA, Avise JC (2000). Microsatellite variation in marine, freshwater and anadromous fishes compared with other animals. J. Fish Biol.

[b19] Dlugosch KM, Parker IM (2008a). Invading populations of an ornamental shrub show rapid life history evolution despite genetic bottlenecks. Ecol. Lett.

[b20] Dlugosch KM, Parker IM (2008b). Founding events in species invasions: genetic variation, adaptive evolution, and the role of multiple introductions. Mol. Ecol.

[b21] Driscoll DA, Felton A, Gibbons P, Felton AM, Munro NT, Lindenmayer DB (2012). Priorities in policy and management when existing biodiversity stressors interact with climate-change. Clim. Change.

[b22] Earl DA, vonHoldt BM (2012). STRUCTURE HARVESTER: a website and program for visualizing STRUCTURE output and implementing the Evanno method. Conserv. Genet. Resour.

[b23] Estoup A, Guillemaud T (2010). Reconstructing routes of invasion using genetic data: why, how and so what?. Mol. Ecol.

[b24] Excoffier L, Lischer HEL (2010). Arlequin suite ver 3.5: a new series of programs to perform population genetics analyses under Linux and Windows. Mol. Ecol. Resour.

[b25] Facon B, Genton BJ, Shykoff J, Jarne P, Estoup A, David P (2006). A general eco-evolutionary framework for understanding bioinvasions. Trends Ecol. Evol.

[b26] Facon B, Pointier J-P, Jarne P, Sarda V, David P (2008). High genetic variance in life-history strategies within invasive populations by way of multiple introductions. Curr. Biol.

[b27] Geller JB, Darling JA, Carlton JT (2010). Genetic perspectives on marine biological invasions. Ann. Rev. Mar. Sci.

[b28] Ghalambor CK, McKay JK, Carroll SP, Reznick DN (2007). Adaptive versus non-adaptive phenotypic plasticity and the potential for contemporary adaptation in new environments. Funct. Ecol.

[b29] Gjelland KØ, Bøhn T, Amundsen PA (2007). Is coexistence mediated by microhabitat segregation? An in-depth exploration of a fish invasion. J. Fish Biol.

[b30] Golubtsov BAS, Ilyin II, Mina MV (1993). Polymorphisms at two enzyme loci (Sod and Odh) in populations of the Amur sleeper, *Perccottus glenii* (Pisces, Eleotrididae), from its native range and the colonized area: the effect of introduction on genetic variation. J. Zoolog. Syst. Evol. Res.

[b31] Guo SW, Thompson EA (1992). Performing the exact test of Hardy-Weinberg proportion for multiple alleles. Biometrics.

[b32] Gutowsky LFG, Fox MG (2012). Intra-population variability of life-history traits and growth during range expansion of the invasive round goby, *Neogobius melanostomus*. Fish. Manage. Ecol.

[b33] Hedrick PW (2000). Genetics of populations.

[b34] Henry P, Goudet G, Le Lay J, Guisan A, Jahodova S, Besnard G (2009). Reduced genetic diversity, increased isolation and multiple introductions of invasive giant hogweed in the western Swiss Alps. Mol. Ecol.

[b35] Hewitt GM (1996). Some genetic consequences of ice ages, and their role in divergence and speciation. Biol. J. Linn. Soc.

[b36] Hubisz MJ, Falush D, Stephens M, Pritchard JK (2009). Inferring weak population structure with the assistance of sample group information. Mol. Ecol. Resour.

[b37] Ibrahim KM, Nichols RA, Hewitt GM (1996). Spatial patterns of genetic variation generated by different forms of dispersal during range expansion. Heredity.

[b38] Kalinowski ST (2005). HP-RARE 1.0: a computer program for performing rarefaction on measures of allelic richness. Mol. Ecol. Notes.

[b39] Knutsen H, Jorde PE, Andre C, Stenseth NC (2003). Fine-scaled geographical population structuring in a highly mobile marine species: the Atlantic cod. Mol. Ecol.

[b40] Kolbe JJ, Glor RE, Schettino LRG, Lara AC, Larson A, Losos JB (2004). Genetic variation increases during biological invasion by a Cuban lizard. Nature.

[b41] Langella O (2005). http://bioinformatics.org/project/?group_id=84.

[b42] Lavergne S, Molofsky J (2007). Increased genetic variation and evolutionary potential drive the success of an invasive grass. Proc. Natl. Acad. Sci. USA.

[b43] Lee CE (2002). Evolutionary genetics of invasive species. Trends Ecol. Evol.

[b44] Lockwood JL, Cassey P, Blackburn T (2005). The role of propagule pressure in explaining species invasions. Trends Ecol. Evol.

[b45] Lodge DM (1993). Biological invasions – lessons for ecology. Trends Ecol. Evol.

[b46] Luikart G, Cornuet JM (1998). Empirical evaluation of a test for identifying recently bottlenecked populations from allele frequency data. Conserv. Biol.

[b47] Mack RN, Simberloff D, Lonsdale WM, Evans H, Clout M, Bazzaz FA (2000). Biotic invasions: causes, epidemiology, global consequences, and control. Ecol. Appl.

[b48] Mariani S, Hutchinson WF, Hatfield EMC, Ruzzante DE, Simmonds EJ, Dahlgren TG (2005). North Sea herring population structure revealed by microsatellite analysis. Mar. Ecol. Prog. Ser.

[b49] Mooney HA, Hobbs RJ (2000). Invasive species in a changing world.

[b50] Moyle PB, Mooney HA, Drake JA (1986). Fish introductions into North America. Ecology of biological invasions of North America and Hawaii.

[b51] Mutenia A, Salonen E (1992). The vendace (*Coregonus albula* L.), a new species in the fish community and fisheries of Lake Inari. Pol. Arch. Hydrobiol.

[b52] Nei M, Maruyama T, Chakraborty R (1975). Bottleneck effect and genetic variability in populations. Evolution.

[b53] Nei M, Tajima F, Tateno Y (1983). Accuracy of estimated phylogenetic trees from molecular-data. 2. Gene-frequency data. J. Mol. Evol.

[b54] Novak SJ, Mack RN, Sax DF, Stachowicz JJ, Gaines SD (2005). Genetic bottlenecks in alien plant species: influences of mating systems and introduction dynamics. Species invasions: insights into ecology, evolution, and biogeography.

[b55] Olden JD, Poff NL (2004). Ecological processes driving biotic homogenization: testing a mechanistic model using fish faunas. Ecology.

[b56] Olden JD, Rooney TP (2006). On defining and quantifying biotic homogenization. Glob. Ecol. Biogeogr.

[b57] Østbye K, Amundsen PA, Bernatchez L, Klemetsen A, Knudsen R, Kristoffersen R (2006). Parallel evolution of ecomorphological traits in the European whitefish *Coregonus lavaretus* (L.) species complex during postglacial times. Mol. Ecol.

[b58] Page RDM (1996). TreeView: an application to display phylogenetic trees on personal computers. Comput. Appl. Biosci.

[b59] Parker IM, Simberloff D, Lonsdale WM, Goodell K, Wonham M, Kareiva PM (1999). Impact: toward a framework for understanding the ecological effects of invaders. Biol. Invasions.

[b60] Peakall R, Smouse PE (2006). GenAlEx 6: genetic analysis in Excel. Population genetic software for teaching and research. Mol. Ecol. Notes.

[b61] Peterson DP, Fausch KD, White GC (2004). Population ecology of an invasion: effects of brook trout on native cutthroat trout. Ecol. Appl.

[b62] Pimentel D, Lach L, Zuniga R, Morrison D (2000). Environmental and economic costs of nonindigenous species in the United States. Bioscience.

[b63] Præbel K, Westgaard J-I, Fevolden S-E, Christiansen JS (2008). Circumpolar genetic population structure of capelin, *Mallotus villosus*. Mar. Ecol. Prog. Ser.

[b64] Præbel K, Westgaard J-I, Amundsen P-A, Siwertsson A, Knudsen R, Kahilainen KK A diagnostic tool for efficient analysis of population structure, hybridization and conservation status of European whitefish (*Coregonus lavaretus* (L.)) and vendace (*C. albula* (L.)). Adv. Limnol.

[b65] Pritchard JK, Stephens M, Donnelly P (2000). Inference of population structure using multilocus genotype data. Genetics.

[b66] Rahel FJ (2000). Homogenization of fish faunas across the United States. Science.

[b67] Rahel FJ (2002). Homogenization of freshwater faunas. Annu. Rev. Ecol. Syst.

[b68] Reusch TBH, Bolte S, Sparwel M, Moss AG, Javidpour J (2010). Microsatellites reveal origin and genetic diversity of Eurasian invasions by one of the world's most notorious marine invader, *Mnemiopsis leidyi* (Ctenophora). Mol. Ecol.

[b69] Rice WR (1989). Analyzing tables of statistical tests. Evolution.

[b70] Rousset F (2007). Genepop'007: a complete reimplementation of the Genepop software for Windows and Linux. Mol. Ecol. Resour.

[b71] Saisa M, Ronn J, Aho T, Bjorklund M, Pasanen P, Koljonen ML (2008). Genetic differentiation among European whitefish ecotypes based on microsatellite data. Hereditas.

[b72] Sakai AK, Allendorf FW, Holt JS, Lodge DM, Molofsky J, With KA (2001). The population biology of invasive species. Annu. Rev. Ecol. Syst.

[b73] Salonen E (1998). The vendace stock and fisheries in lake Inari. Boreal Environ. Res.

[b74] Salonen E (2004). Estimation of vendace year-class strength with different methods in the subarctic lake Inari. Ann. Zool. Fenn.

[b75] Salonen E, Amundsen P-A, Bøhn T (2007). Boom and bust development by invading vendace *Coregonus albula* in the subarctic lnari-Pasvik watershed (Finland, Norway and Russia). Adv. Limnol.

[b76] Sandlund OT (1992). Differences in the ecology of two vendace populations separated in 1895. Nord. J. Freshw. Res.

[b77] Sandlund OT, Schei PJ, Viken Å (1999). Invasive species and biodiversity management.

[b78] Sax DF, Stachowicz JJ, Brown JH, Bruno JF, Dawson MN, Gaines SD (2007). Ecological and evolutionary insights from species invasions. Trends Ecol. Evol.

[b79] Shea K, Chesson P (2002). Community ecology theory as a framework for biological invasions. Trends Ecol. Evol.

[b80] Shine R (2012). Invasive species as drivers of evolutionary change: cane toads in tropical Australia. Evol. Appl.

[b81] Simberloff D (2009). The role of propagule pressure in biological invasions. Annual review of ecology, evolution and systematics.

[b82] Stepien CA, Brown JE, Neilson ME, Tumeo MA (2005). Genetic diversity of invasive species in the Great Lakes versus their Eurasian source populations: insights for risk analysis. Risk Anal.

[b83] Suarez AV, Tsutsui ND (2008). The evolutionary consequences of biological invasions. Mol. Ecol.

[b84] Szpiech ZA, Jakobsson M, Rosenberg NA (2008). ADZE: a rarefaction approach for counting alleles private to combinations of populations. Bioinformatics.

[b85] Tallmon DA, Koyuk A, Luikart G, Beaumont MA (2008). ONeSAMP: a program to estimate effective population size using approximate Bayesian computation. Mol. Ecol. Resour.

[b86] Tonione MA, Reeder N, Moritz CC (2011). High genetic diversity despite the potential for stepping-stone colonizations in an invasive species of gecko on Moorea, French Polynesia. PLoS ONE.

[b87] Tsutsui ND, Suarez AV, Holway DA, Case TJ (2000). Reduced genetic variation and the success of an invasive species. Proc. Natl. Acad. Sci. USA.

[b88] Van Oosterhout C, Hutchinson WF, Wills DPM, Shipley P (2004). MICRO-CHECKER: software for identifying and correcting genotyping errors in microsatellite data. Mol. Ecol. Notes.

[b89] Vitousek PM, DAntonio CM, Loope LL, Westbrooks R (1996). Biological invasions as global environmental change. Am. Sci.

[b90] Ward JL, Blum MJ, Walters DM, Porter BA, Burkhead N, Freeman B (2012). Discordant introgression in a rapidly expanding hybrid swarm. Evol. Appl.

[b91] Wares JP, Hughes AR, Grosberg RK, Sax DF, Stachowicz JJ, Gaines SD (2005). Mechanisms that drive evolutionary change: insights from species introductions and invasions. Species invasions: insights into ecology, evolution, and biogeography.

[b92] Weir BS, Cockerham CC (1984). Estimating F-statistics for the analysis of population-structure. Evolution.

[b93] Williamson M (1996). Biological invasions.

[b94] Wilson JRU, Dormontt EE, Prentis PJ, Lowe AJ, Richardson DM (2009). Something in the way you move: dispersal pathways affect invasion success. Trends Ecol. Evol.

