# Supplementary material for: Invasion genetics of vendace (Coregonus albula (L.)) in the Inari-Pasvik watercourse: revealing the origin and expansion pattern of a rapid colonization event
Source: Ecol Evol. 2013 Apr 12;3(5):1400–12. doi: 10.1002/ece3.552 (PMC3678492; doi:10.1002/ece3.552)
Supplement: Supplementary file 3 [file ece30003-1400-SD3.pdf]

Supplementary material

Table S1 Summary of sample sizes (N), number of alleles (N_A), observed (H_o) and expected (H_e) heterozygosity, and F_{IS}-estimates of the vendace populations at 10 microsatellite loci. F_{IS} * indicate possible null alleles estimated by MICROCHECKER. Underscored and bold F_{IS} values indicate significant departure from HWE before and after sequential Bonferroni corrections, respectively. Sample abbreviations as in Table 1.

Pop		BFRO-018[1]	BWF1[2]	ClaTet06[3]	ClaTet13[3]	Cocl-lav04[4]	Cocl-lav06[4]	Cocl-lav10[4]	Cocl-lav27[4]	Cocl-lav49[4]	C2-157[5]
Ke	N	52	52	52	50	52	52	51	52	52	52
	N_A	2	5	6	4	3	7	4	2	12	2
	H_o	0.019	0.442	0.462	0.520	0.288	0.654	0.490	0.038	0.712	0.135
	H_e	0.019	0.365	0.615	0.576	0.302	0.674	0.588	0.038	0.708	0.126
	F_{IS}	-0.010	-0.212	0.249*	0.097	0.045	0.030	0.167	-0.020	0.034	-0.072
Si	N	57	58	57	57	58	57	57	58	55	58
	N_A	7	13	6	16	2	19	3	3	12	8
	H_o	0.702	0.741	0.579	0.807	0.293	0.754	0.211	0.121	0.782	0.448
	H_e	0.594	0.796	0.674	0.896	0.274	0.874	0.610	0.114	0.806	0.449
	F_{IS}	-0.181	0.068	0.141	0.099*	-0.070	0.137*	**0.655***	-0.056	0.031	0.001
In	N	55	52	55	54	55	52	51	55	55	55
	N_A	5	11	5	16	2	25	4	4	11	6
	H_o	0.473	0.692	0.491	0.889	0.309	0.808	0.314	0.073	0.782	0.636
	H_e	0.469	0.738	0.573	0.898	0.261	0.916	0.596	0.071	0.808	0.613
	F_{IS}	-0.007	0.062	0.143	0.010	-0.183	0.119*	**0.474***	-0.026	0.033	-0.037
Vg	N	57	57	57	57	57	57	57	57	57	57

	N_A	7	9	5	15	3	22	3	3	8	6
	H_o	0.474	0.632	0.561	0.895	0.246	0.807	0.404	0.088	0.754	0.596
	H_e	0.482	0.743	0.603	0.878	0.217	0.913	0.600	0.085	0.812	0.572
	F_{IS}	0.017	0.149*	0.068	-0.019	-0.130	**0.116***	**0.327***	-0.034	0.071	-0.042
Sb	N	54	53	54	53	54	54	54	53	54	54
	N_A	6	10	6	16	2	19	3	3	8	6
	H_o	0.500	0.491	0.574	0.717	0.389	0.778	0.333	0.094	0.630	0.500
	H_e	0.431	0.716	0.661	0.873	0.335	0.899	0.501	0.091	0.784	0.604
	F_{IS}	-0.159	**0.315***	0.132	**0.178***	-0.160	0.135*	**0.335***	-0.037	0.197*	0.173*

[1] Susnik et al. 1999; [2] Patton et al. 1997; [3] Winkler & Weiss 2008; [4] Rogers et al. 2004; [5] Turgeon et al. 1999

Table S2 Geographical distances and expansion times of vendace populations used for the Mantel test. Sample abbreviations as in Table 1.

	Geographical distance (km)	Time (years)		
		Initial_observation	Total_coverage	Peak_abundance
Si_In	302	8	8	8
Si_Vgt	334	26	26	26
Si_Sb	383	28	28	28
In_Vgt	47	18	11	2
In_Sb	95	20	13	4
Vgt_Sb	51	2	2	2

Table S3 Estimates of genetic differentiation between the five populations of vendace revealed by F_{ST} (Below diagonal) and the permutated (10,000) *P*-values (above diagonal). Significance levels (*P*-values) were adjusted according to the sequential Bonferroni procedure (Rice 1989). Sample codes as in Table I.

	Ke	Si	In	Vg	Sb
Ke	-	0.0001	0.0001	0.0001	0.0001
Si	0.141	-	0.0027	0.0013	0.0014
In	0.158	0.011	-	0.0015	0.0032
Vg	0.154	0.011	0.011	-	0.1603
Sb	0.157	0.012	0.011	0.004	_

Fig. S1. Summary of results from STRUCTURE for inferring K populations among the tested vendace populations when omitting three loci that may be associated with null alleles. The most likely grouping was estimated by 5 independent runs for each K (A). In B the genetic structure among L. Kelujärvi (Ke). L. Sinettäjärvi (Si). L. Inari (In), L. Vaggatem (Vg) and L. Skrukkebukta (Sb) was tested using the same set of seven loci and Bayesian clustering assuming two genetic clusters of individuals (K = 2). In the STRUCTURE analysis black lines separate individuals from different sampling sites and each individual is represented by a thin horizontal line, which is partitioned into K-coloured segments representing individual's estimated membership fractions in K clusters.

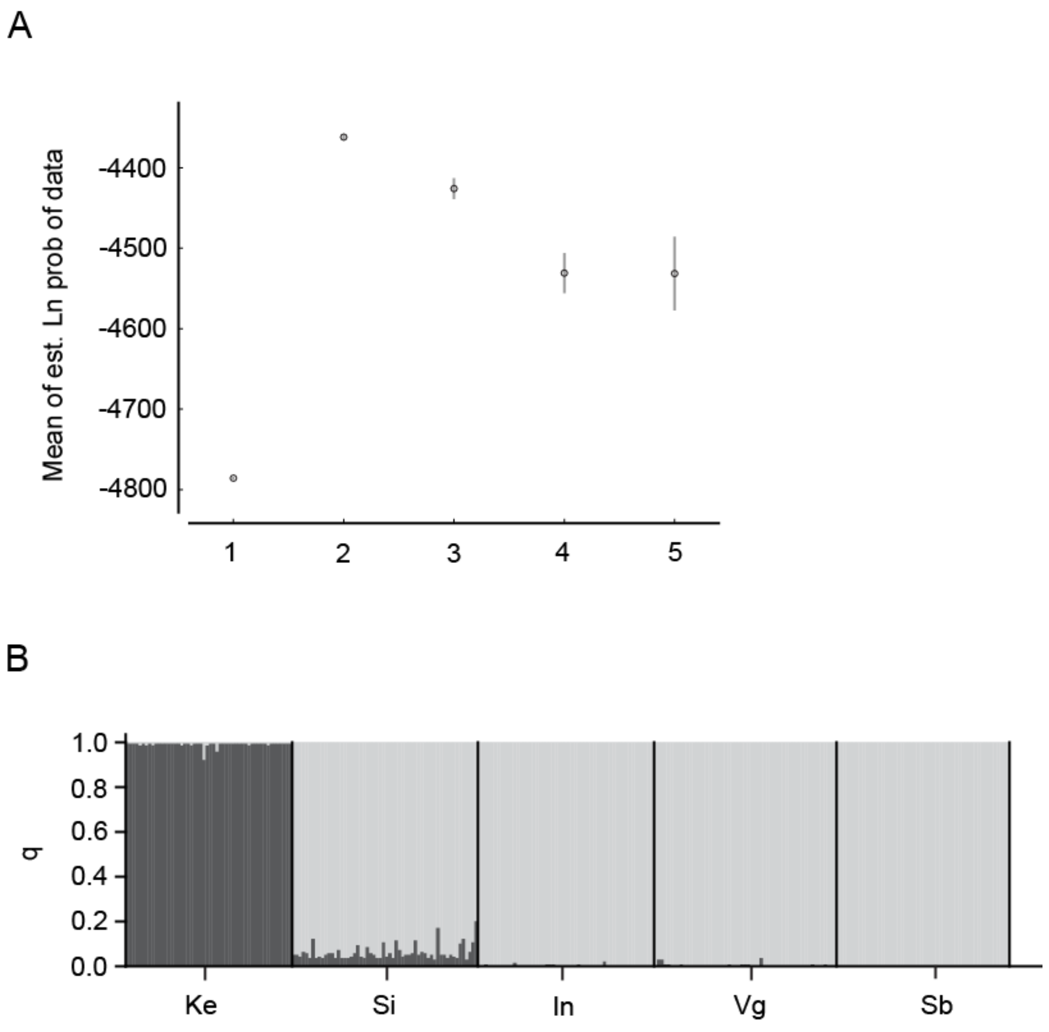

Fig. S2 Summary of results from STRUCTURE for inferring K populations among the tested vendace populations. The most likely grouping was estimated by 10 independent runs for each K.

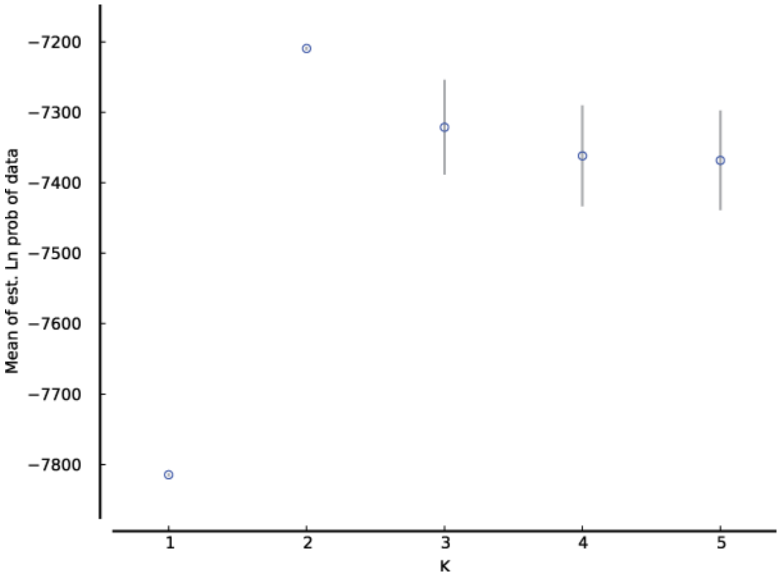

References

Patton JC, Gallaway BJ, Fechhelm RG, Cronin MA (1997) Genetic variation of microsatellite and mitochondrial DNA markers in broad whitefish (*Coregonus nasus*) in the Colville and Sagavanirktok rivers in northern Alaska. *Canadian Journal of Fisheries and Aquatic Sciences* **54**, 1548-1556.

Rogers SM, Marchand MH, Bernatchez L (2004) Isolation, characterization and cross-salmonid amplification of 31 microsatellite loci in the lake whitefish (*Coregonus clupeaformis*, Mitchill). *Molecular Ecology Notes* **4**, 89-92.

Susnik S, Snoj A, Dovc P (1999) Microsatellites in grayling (*Thymallus thymallus*): comparison of two geographically remote populations from the Danubian and Adriatic river basin in Slovenia. *Molecular Ecology* **8**, 1756-1758.

Turgeon J, Estoup A, Bernatchez L (1999) Species flock in the North American Great Lakes: Molecular ecology of Lake Nipigon Ciscoes (Teleostei : Coregonidae : Coregonus). *Evolution* **53**, 1857-1871.

Winkler KA, Weiss S (2008) Eighteen new tetranucleotide microsatellite DNA markers for *Coregonus lavaretus* cloned from an alpine lake population. *Molecular Ecology Resources* **8**, 1055-1058.
